# Differential networking meta-analysis of gastric cancer across Asian and American racial groups

**DOI:** 10.1186/s12918-018-0564-z

**Published:** 2018-04-24

**Authors:** Wentao Dai, Quanxue Li, Bing-Ya Liu, Yi-Xue Li, Yuan-Yuan Li

**Affiliations:** 1 Shanghai Center for Bioinformation Technology, 1278 Keyuan Road, Shanghai, 201203 People’s Republic of China; 2 School of biotechnology, East China University of Science and Technology, Shanghai, 200237 China; 3 Shanghai Key Laboratory of Gastric Neoplasms, Shanghai Institute of Digestive Surgery, Ruijin Hospital, Shanghai Jiao Tong University School of Medicine, Shanghai, 200025 People’s Republic of China; 4 Shanghai Engineering Research Center of Pharmaceutical Translation & Shanghai Industrial Technology Institute, 1278 Keyuan Road, Shanghai, 201203 People’s Republic of China; 5 Key Lab of Computational Biology, CAS-MPG Partner Institute for Computational Biology, Shanghai Institutes for Biological Sciences, Chinese Academy of Sciences, Shanghai, 200031 China

**Keywords:** Gastric carcinoma (GC), Differential networking meta-analysis, Cross-racial, Conditional gene regulatory networks (GRN), Dysfunctional regulation mechanisms

## Abstract

**Background:**

Gastric Carcinoma is one of the most lethal cancer around the world, and is also the most common cancers in Eastern Asia. A lot of differentially expressed genes have been detected as being associated with Gastric Carcinoma (GC) progression, however, little is known about the underlying dysfunctional regulation mechanisms. To address this problem, we previously developed a differential networking approach that is characterized by involving differential coexpression analysis (DCEA), stage-specific gene regulatory network (GRN) modelling and differential regulation networking (DRN) analysis.

**Result:**

In order to implement differential networking meta-analysis, we developed a novel framework which integrated the following steps. Considering the complexity and diversity of gastric carcinogenesis, we first collected three datasets (GSE54129, GSE24375 and TCGA-STAD) for Chinese, Korean and American, and aimed to investigate the common dysregulation mechanisms of gastric carcinogenesis across racial groups. Then, we constructed conditional GRNs for gastric cancer corresponding to normal and carcinoma, and prioritized differentially regulated genes (DRGs) and gene links (DRLs) from three datasets separately by using our previously developed differential networking method. Based on our integrated differential regulation information from three datasets and prior knowledge (e.g., transcription factor (TF)-target regulatory relationships and known signaling pathways), we eventually generated testable hypotheses on the regulation mechanisms of two genes, XBP1 and GIF, out of 16 common cross-racial DRGs in gastric carcinogenesis.

**Conclusion:**

The current cross-racial integrative study from the viewpoint of differential regulation networking provided useful clues for understanding the common dysfunctional regulation mechanisms of gastric cancer progression and discovering new universal drug targets or biomarkers for gastric cancer.

**Electronic supplementary material:**

The online version of this article (10.1186/s12918-018-0564-z) contains supplementary material, which is available to authorized users.

## Background

Gastric Carcinoma (GC) is one of the most common and lethal tumor around the world, which is characterized by high heterogeneity, easy metastasis, and poor prognosis [1, 2]. The morbidity and mortality of gastric carcinoma in Eastern Asia are much higher than the world average level [3]. During the last decade, quite a series of high-throughput profiling, including genetic variation study [4–7], genome-wide association study (GWAS) [7], gene expression analysis [8–10], epigenetic variation study [11], and integrative genomic analysis [12–15], have greatly help to understand the biology of GC, and identified quite a lot of GC-associated genes. It was noticed that the above studies always collected GC samples from a certain racial group such as Chinese [7, 12, 16], Korean [8], and American [14, 17], respectively, while the common dysregulation mechanisms of gastric carcinogenesis across racial groups has been paid little attention due to lack of integration research based on cross-racial GC datasets.

It has been widely accepted that cancer results from the dysregulation of multiple fundamental cell processes including proliferation, differentiation, migration, apoptosis, and so on [18], which could be captured by gene regulatory network (GRN) modelling, a widely used approach to explore the pathogenesis of complex diseases from the systemic aspect [19–21]. In recent years, a novel theme “differential networking” was put forward and a number of methods have been developed to identify the regulators, the relationships, and even the sub-networks that differ between phenotypes [20–23]. With the rapid accumulation of transcriptomic data, differential network analysis is helpful to survey the dynamics of gene regulation, which is crucial to the understanding of pathophysiological processes [24–26]. In our previous studies, we designed and implemented a differential co-expression analysis (DCEA) approach called DCGL to recognize differential co-expression genes (DCGs) and links (DCLs) in a link-based quantitative way [27–29]. Based on this methodology, we further developed a differential regulation networking (DRN) framework [30, 31], which built conditional gene regulatory network (GRN) or combinatorial GRN (cGRN) and then prioritized differentially regulated genes (DRGs) and links (DRLs). Our DRN strategy proves to substantially reduce the computational burden and leads to insightful comments on selecting subject related genes and their differential regulation mechanisms underlying phenotypic changes.

Based on the previous methodologies, the current study aimed at investigating the common dysregulation mechanisms of gastric carcinogenesis across Chinese, Korean and American. To this end, we constructed a novel integrative analysis framework from the viewpoint of differential regulation, which integrated a variety of modules with differential regulation networking (DRN) analysis and integrative analysis as the core steps. First of all, the conditional gene regulation networks (GRN) were built from three Gastric Carcinoma datasets (GSE54129, GSE24375 and TCGA-STAD) separately, and differentially regulated genes (DRGs) and differentially regulated links (DRLs) were prioritized then. It was found that known cancer genes and drug targets are significantly ranked higher, and most of top-10 DRGs from the three datasets have been reported to be GC related (~ 60%), or cancer related (~ 90%); meanwhile, there is a lack of consistency among the three top DRG lists. By integrating DRGs and DRLs from three datasets to the prior regulation knowledge, it was found that the 16 common DRGs across racial groups are mainly located in the transcription factor complex in nucleus and their functions were enriched in transcriptional regulation of RNA polymerase II, transcriptional activator activity and transcription factor binding. We therefore proposed two common cross-racial DRGs (GIF, XBP1) and their related regulation relationships which might play crucial roles in the dysregulation mechanisms of gastric carcinogenesis. This integration analysis of GC across racial groups provided useful clues for understanding common dysfunctional regulation mechanisms of gastric carcinogenesis and discovering new universal drug targets or biomarkers for gastric cancer, and also indicated the complexity and diversity of gastric carcinogenesis as well.

## Methods

### Gene expression datasets

The Affymetrix GeneChip Operating System (GCOS) was used to measure expression level of 111 Chinese gastric carcinoma samples and 21 Chinese normal mucosa samples (GSE54129). The raw expression datasets were normalized by robust RMA method and log2 transformed. The evaluation of GSE54129 raw data in terms of expression level distribution, density distribution and correlations of samples were shown in Additional file 1: Figure S1. After mapping probe sets to Gene Symbols based on their platform annotations, 20,307 unique genes were obtained.

We also downloaded the mRNA expression dataset of stomach adenocarcinoma (STAD) from The Cancer Genome Atlas (TCGA) Data Protal (https://cancergenome.nih.gov/), which contains sequenced 29 matched American tumor-normal pairs with Illumina Hiseq platform. After discarding genes with more than 20% missing values, we got 19,211 RPKM normalized and log2 transformed unique genes.

The normalized gene expression profile of Korean gastric carcinoma GSE24375 [7] was downloaded from Gene Expression Omnibus (GEO) and all measurements were log2 transformed. The dataset includes eight patient-matched gastric normal mucosa, adenoma and carcinoma samples and two additional carcinoma samples. Probe sets with more than 20% missing values were discarded, while probe sets with less missing values were filled up with KNN method. After probe sets filtering, 18,468 probe sets were mapped to Gene Symbols based on their platform annotations and 12,658 unique genes were obtained. The distributions of normalized and log2 transformed expression levels of genes in three datasets are presented in Additional file 1: Figure S2.

### Enrichment analysis: Function, pathway, cancer genes and drug targets

The Database for Annotation, Visualization and Integrated Discovery (DAVID) [32] was used to identify over-represented KEGG pathways and GO terms based on the hypergeometric distribution with *p*-values < 0.05 were considered statistically significant.

A total of 486 cancer genes and 2093 drug targets were downloaded from Cancer Gene Census (http://cancer.sanger.ac.uk/cancergenome/projects/census/) and DrugBank (http://www.drugbank.ca/), respectively, which were used to validate the differential regulation analysis on the three gene expression datasets.

### Differential networking meta-analysis framework

In order to implement differential networking meta-analysis, we developed a novel framework which integrated a variety of modules as follows (outlined in Additional file 1: Figure S3).

Differentially co-expressed genes (DCGs) and links (DCLs) were identified with our previously developed differential coexpression analysis (DCEA) methods [27, 28]. DCGs with *p-*values less than 0.05 were selected by DCp method, and DCLs were picked out by DCe method with LFC model in DCGL package [28].

The conditional gene regulatory networks (GRN) were constructed for the three preprocessed datasets respectively based on DCGs and DCLs by using the conditional GRN modelling approach developed in our previous work [30]. First, we applied DCGL package to the expression dataset (GSE54129, GSE24375 and TCGA-STAD, respectively) to extract differentially coexpressed genes (DCGs) and differentially coexpressed gene links (DCLs) between normal and cancer. The DCGs and the gene pairs in the DCLs which involved at least one DCG between normal and carcinoma were selected as core seeds for the construction of conditional GRNs. Then we constructed the conditional GRNs based on forward predicted TF-target regulatory relationships and the core seed genes by using stepwise linear regression according to our previous method [30]. In this way, we built three normal and cancer GRN pairs corresponding to the three expression datasets (GSE54129, GSE24375 and TCGA-STAD) separately.

The differential regulated genes (DRGs) and links (DRLs) in conditional GRNs were ranked by our previously developed quantitatively methods [30], DR measure and modified LFC model, respectively. The power of the above two methods in cancer genes and drug targets prioritization have been validated strictly in our previous work. In order to further test the power of prioritization of DRGs and DRLs methods in the three GRN pairs from 3 GC gene expression datasets, we carried out permutation tests by randomly perturbing the DRGs lists for 5000 times for each gene expression dataset as similar as the method described previously [30, 33].

The integrative analysis was carried out based on both genes and functions. In order to prioritize common DRGs across gastric cancer datasets, we combined DRG lists from the three GC datasets to select cross-racial DRGs which listed in the top N (< 100) DRGs commonly in at least two racial groups. To globally understand the common functional relevance of differential regulation across all the three GC datasets, we performed GO/KEGG functional enrichment analysis on both top 10 DRGs out of every single dataset and common cross-racial DRGs by using DAVID 6.8.

## Results

### Screening for differentially co-expressed genes

According to DCGL method [28], the genes with the *p*-value of dC less than 0.05 were taken as DCGs between normal and carcinoma. A total of 3875 DCGs were selected from GSE54129 dataset; 3604 DCGs from TCGA-STAD dataset; and 2524 DCGs from GSE24375 dataset.

**Fig. 1:**
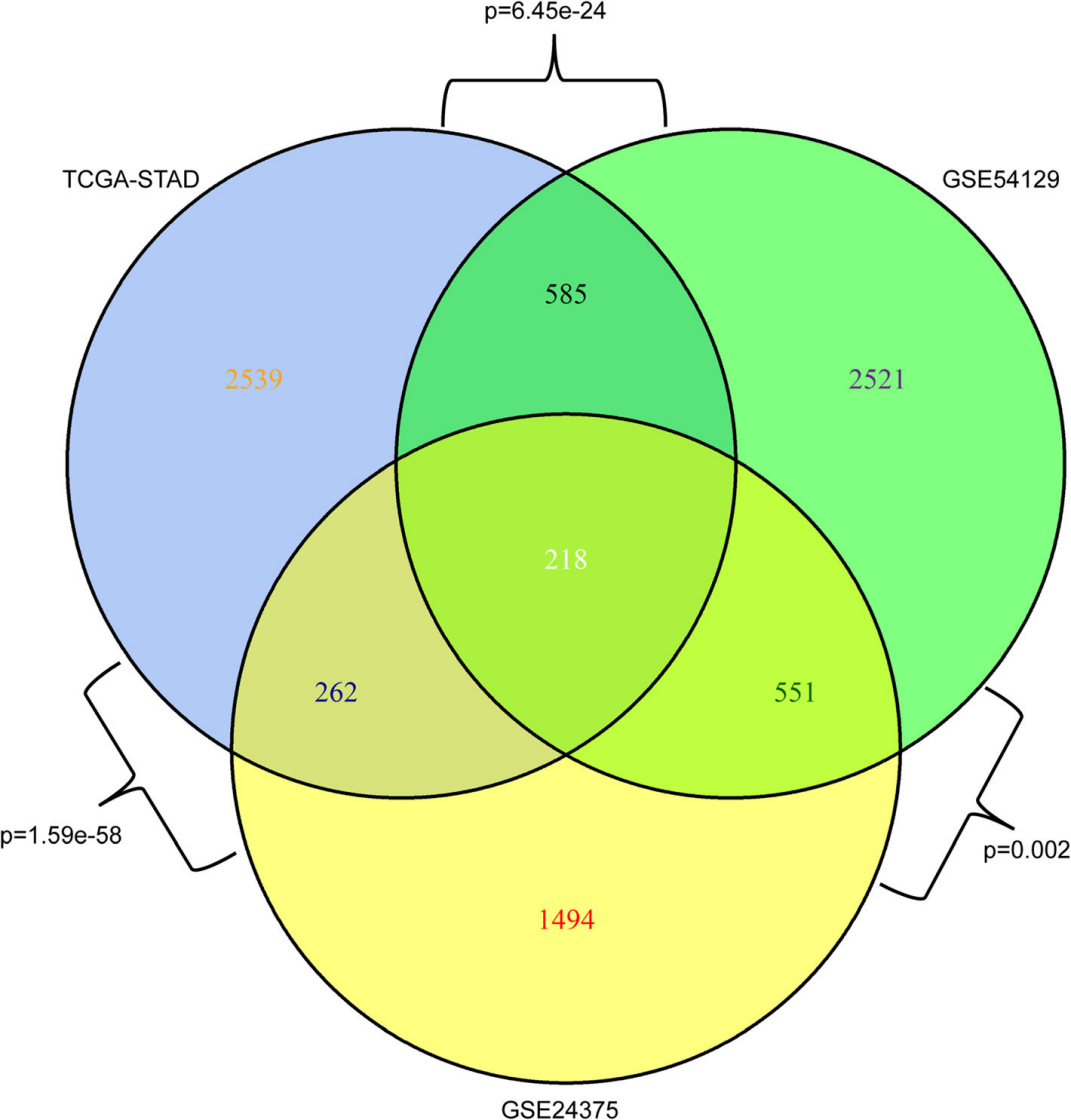
The intersection and enrichment significance among three DCG sets selected from GSE54129 (Chinese, yellow), GSE24375 (Korean, green) and TCGA-STAD (American, cyan) gastric cancer datasets

The intersection and enrichment significance among three DCG sets (Fig. 1) shows that the DCGs between every two datasets are significantly overlapped by Kappa test. This suggests a basically coherent profile of molecular interactions underlying gastric carcinogenesis across racial groups included in this study, Chinese, Korean, and American.

### Construction of GRNs and identification of DRGs and DRLs

**Fig. 2:**
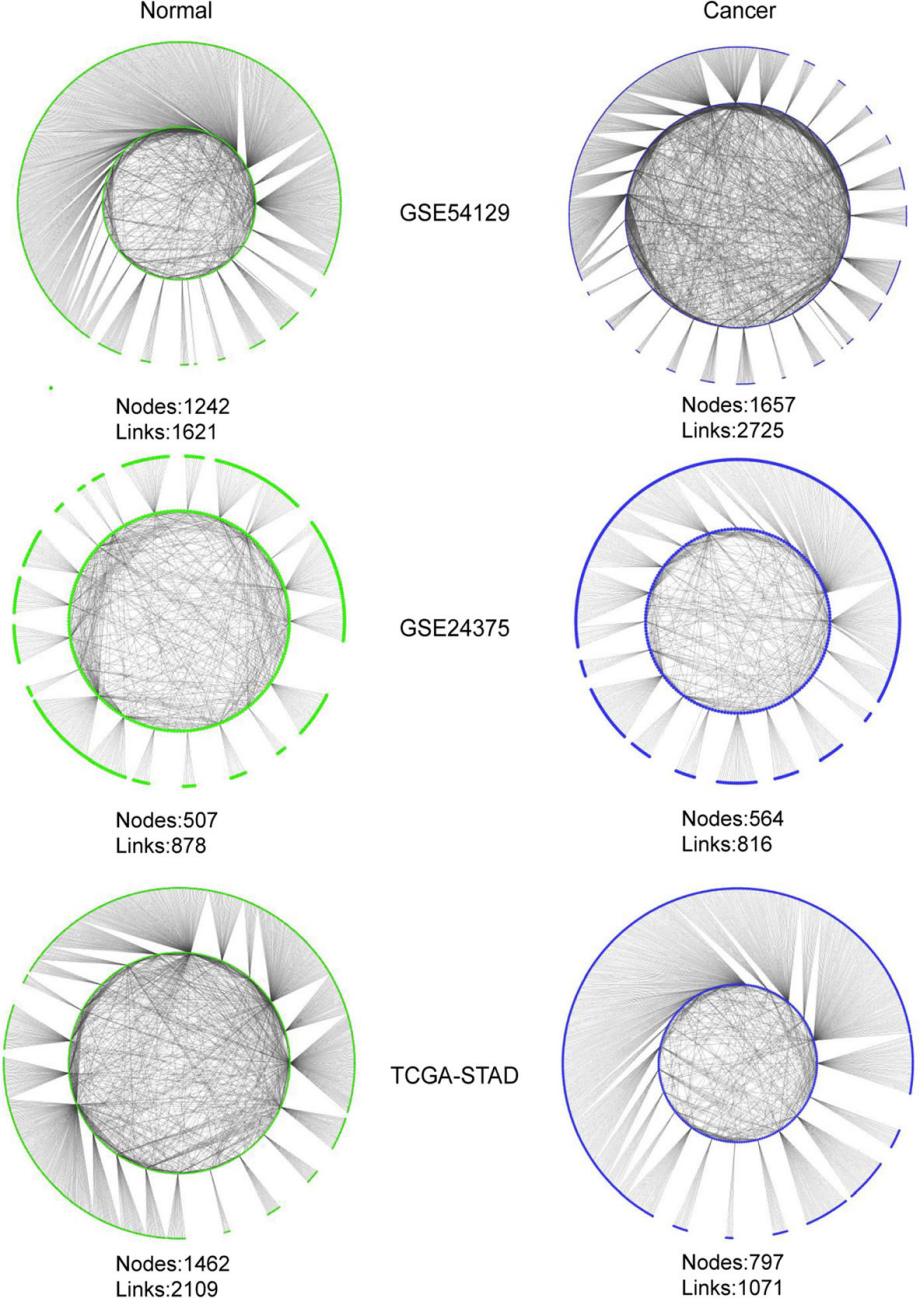
The normal and carcinoma conditional gene regulation networks (GRNs) for GSE54129 (Chinese), GSE24375 (Korean) and TCGA-STAD (American) gastric cancer datasets

Based on the (normal and carcinoma) expression data of the selected DCG sets, we built paired conditional (normal and carcinoma) GRNs (Fig. 2) respectively corresponding to Chinese (GSE54129), Korean (GSE24375) and American (TCGA-STAD) by using the method described in the section of Materials and methods. The statistics of the three pairs of GRNs are listed in Table 1.It was found that the paired GRNs, i.e., normal and carcinoma GRNs for a certain dataset, always share the same regulators and most of the target genes, however, they share only a few of links, indicating that our analysis method did efficiently narrow down the search space and build stage-specific networks enriching subject relevant regulation relationships as expected [30, 31]. We then checked the global topological change between normal and cancer GRNs across the three datasets. According to the node number, the network size for Chinese (GSE54129) and Korean (GSE24375) expanded from normal to cancer, while that for American (TCGA-STAD) shrank. According to the average degree of nodes, the network complexity for Chinese (GSE54129) increased from normal to cancer, while that for Korean (GSE24375) and American (TCGA-STAD) decreased. Considering the inconsistency of the sample size of the three datasets, the above observation need to be further evaluated based on data from larger patient populations.

**Table 1:** Statistics of conditional GRNs from three datasets

	GSE54129			TCGA-STAD			GSE24375		
GRNs	Links (#)	TFs (#)	Targets (#)	Links (#)	TFs (#)	Targets (#)	Links (#)	TFs (#)	Targets (#)
Normal	1621	30	1242	2109	21	1462	878	22	507
Carcinoma	2725	30	1657	1071	21	797	816	22	564
shared by normal and carcinoma GRNs	723	30	972	760	21	718	303	22	331

As shown in Table 2, known cancer genes and drug targets were enriched in all the conditional GRNs by Fisher’s Exact Test, demonstrating that our conditional GRNs have the potential to highlight crucial cancer-related regulation relationships, thus proving the rationality of the three GRN pairs.

**Table 2:** The enrichment of cancer genes and drug targets in conditional GRNs

	GSE54129		TCGA		GSE24375	
GRNs	Cancer Genes	Drug Targets	Cancer Genes	Drug Targets	Cancer Genes	Drug Targets
Normal	8.40E-10	2.84E-13	0.00012	6.776e-08	0.0034	0.039
Carcinoma	2.48E-12	2.21E-10	2.08E-6	4.841e-07	0.0015	0.0002

Enrichment significance (*p*-value) was calculated by Fisher Exact Test

After constructing conditional GRNs, a key issue is to quantitatively analyze the dynamic changes of gene regulation during phenotypic changes, i.e., from normal to carcinoma in the current work. Two methods were used to measure the differential regulation of a specific gene or gene link between two conditional GRNs as described in the section of Materials and methods, based on which differentially regulated genes (DRGs) and differentially regulated gene pairs or links (DRLs) were prioritized.

Permutation test showed that gastric cancer genes were significantly ranked higher in the DRG lists from three GC expression datasets, with *p*-values of 1.36e-05 (GSE24375), 6.54e-53 (GSE54129) and 0.00793 (TCGA-STAD). The rank of drug targets in these DRG lists presented a similar trend, though not significant in datasets GSE24375 and TCGA-STAD. This indicates that DR ranking is appropriate to prioritize disease related genes in the current conditional GRNs.

The top 10 DRGs identified from three GC expression datasets were listed in Table 3. Among the top 10 DRGs for Chinese (GSE54129), six genes have been reported to be gastric cancer (GC) related (REG4 [34], ANXA13 [35], C7 [36], ASS1 [37], MSMB [38], CREB1 [39]) and the rest four were directly regulated by known gastric cancer genes in our GRNs, in which two genes are also cancer related (BPTF [40], CLCA1 [41]). Among the top 10 DRGs identified for Korean (GSE24375), six genes are GC related (ESRRG [42], LIMS1 [43, 44], GATA3 [45], GATA6 [46], SOX9 [47], POU2F1 [48]) and the other four are cancer related (IRF2 [49], RGS3 [50], MRPL36 [51], FOSB [52]). Among the top 10 DRGs identified from TCGA-STAD dataset, six are GC related (E2F1 [50], AK1B10 [51], CEBPA [52], PTK7 [53], RASAL1 [54], GKN [55]), and three are cancer related (DPCR1 [56], GGT6 [57], RAB25 [58]). Additionally, 873 DRLs, 590 DRLs and 319 DRLs were identified for Chinese (GSE54129), American (TCGA-STAD) and Korean (GSE24375), respectively (Additional file 2: Table S1). We noticed that the vast majority of top 10 DRGs are gastric cancer related in all three datasets, while the three gene lists do not overlap at all, suggesting that the most crucial factors during gastric carcinogenesis may vary across racial groups. We therefore carried out the following integrative analysis aiming to identify cross-racial dysregulation mechanisms underlying gastric carcinogenesis.

**Table 3:** The top 10 ranked genes from three datasets

TCGA-STAD			GSE54129			GSE24375		
DRGs	DR_value	rank	DRGs	DR_value	rank	DRGs	DR_value	rank
**E2F1**	18.45799	1	SLC7A9	65.03438	1	**LIMS1**	3401.341	1
**AKR1B10**	15.58216	2	**REG4**	22.26006	2	*FOSB*	582.8947	2
**CEBPA**	12.34533	3	*BPTF*	20.02962	3	*MRPL36*	261.5739	3
**PTK7**	11.85102	4	DHRS11	19.11607	4	**ESRRG**	247.57	4
PLAU	11.5865	5	**ANXA13**	17.92791	5	**GATA3**	132.0578	5
*DPCR1*	11.37501	6	**C7**	15.36403	6	**GATA6**	85.31825	6
*GGT6*	11.14235	7	**ASS1**	15.10191	7	**SOX9**	47.91384	7
RAB25	9.645954	8	**MSMB**	13.03145	8	**POU2F1**	41.98352	8
**RASAL1**	9.191246	9	*CLCA1*	12.91625	9	*IRF2*	37.68406	9
**GKN2**	8.602126	10	**CREB1**	12.84939	10	*RGS3*	35.60814	10

The genes are sorted by DR value. Genes in bold refer to GC-related genes; genes in italic refer to cancer-related genes

### Integrative analysis of cross-racial DRGs and the proposed mechanisms

In order to prioritize common DRGs across gastric cancer datasets, we combined the three DRG lists from the three GC datasets in Table 4. Since there are no common DRGs within the top 50 DRG lists among the three datasets, we first checked the overlap between every two datasets. As shown in Table 4, CEBPA, GATA6, GATA3 and GIF genes appear in the top 30 DRGs commonly in at least two different GC datasets, i.e., two racial groups; similarly, GIF, XBP1, CEBPA, GATA6, DPP4, PTK7 and GATA3 appear in the top 50 DRGs. There are 16 cross-racial DRGs in the top 100 DRGs commonly in at least two racial groups, including GIF, XBP1, CEBPA, GATA6, DPP4, PTK7, GATA3, FOSB, EPAS1, CCND2, MALL, IRF1, SOX9, VILL, GALNT3 and LGALS4. The number of TOP N (<=100) overlapping DRGs across racial groups are not significantly different as shown in Table 4. Meanwhile, we also noticed that among a total number of 6399 DCGs for Chinese and Korean, 12% (769) were shared by the two DCG lists; among 7479 DCG for American and Korean, 10.7% (803) were shared; while among 6128 DCG for Chinese and American, 7.8% (480) were shared (Fig. 1). This suggests that Chinese and Korean have similar gastric carcinogenesis, compared with American.

**Table 4:** Intersection of top ranked DRGs between every two datasets

	Top10	Top20	Top30	Top40	Top50	Top100
**TCGA-STAD vs GSE54129**	0	CEBPA	**CEBPA**	CEBPA	CEBPA	CEBPA/GIF/XBP1
**GSE54129 vs GSE24375**	0	GATA6	**GATA6**	GATA6	GATA6/DPP4	GATA6/GIF/FOSB/XBP1/DPP4/EPAS1/CCND2/MALL
**TCGA-STAD vs GSE24375**	0	0	**GATA3/GIF**	GIF/GATA3/PTK7	GIF/GATA3/PTK7/XBP1	GIF/GATA3/PTK7/XBP1/IRF1/SOX9/VILL/GALNT3/LGALS4
**Across 3 datasets**	0	0	0	0	0	**GIF/XBP1**

GSE54129, GSE24375 and TCGA-STAD collected Chinese, Korean and American gastric cancer samples respectively. The overlaps of top ranked differentially regulated genes (DRGs) between any two datasets (Row 2nd, 3rd and 4th) and between all three datasets (Row 5th) are listed

To globally understand the common functional relevance of differential regulation across the three GC datasets, we performed GO/KEGG functional enrichment analysis on the top ranked DRGs (as shown in Tables 3 and 4) by using DAVID 6.8 [32]. First, the TOP 10 DRGs from three GC dataset (GSE54129, GSE24375 and TCGA-STAD) were analyzed separately. However, no GO/KEGG terms were significantly enriched in any of the three gene sets after multiple hypothesis test correction. Secondly, the terms “transcription regulatory region DNA binding” and “RNA polymerase II transcription factor binding” were identified based on seven common TOP-50 DRGs (GIF, XBP1, CEBPA, GATA6, DPP4, PTK7 and GATA3) between at least two GC datasets. Similarly, based on 16 common TOP-100 DRGs, the terms of “transcription from RNA polymerase II promoter”, “positive regulation of transcription from RNA polymerase II promoter”, “transcriptional activator activity, RNA polymerase II core promoter proximal region sequence-specific binding”, “sequence-specific DNA binding”, “transcription factor complex” and “nucleoplasm” were recognized. That is, the 16 common DRGs across racial groups are mainly located in the transcription factor complex in nucleus and their functions were enriches in transcriptional regulation of RNA polymerase II, transcriptional activator activity and transcription factor binding. This is consistent with the basic understanding that transcription factors (TFs) play crucial roles in the proliferation and differentiation of cells.

It is interesting that GIF, the overlapping gene in the three top-100 gene lists across Chinese, Korean, and American, which is also a TOP-30 DRG commonly in at least two racial groups, was reported to be a prognosis biomarker of gastric cancer and its decreased expression was reported to be correlated with the progresses of gastritis [53, 54]. The down-expression of GIF in gastric carcinoma samples was indeed observed in all three GC dataset. We proposed GIF might be a common GC related gene across racial groups, which participates in carcinogenesis by differential regulation. We then focused on GIF and its surrounding DRLs to generate hypotheses on the regulation mechanisms of gastric carcinogenesis. In our integrative DRN analysis, GIF were regulated by GATA3 in GSE24375 (− 2.896 vs 0.01; DRL TOP 25) and TCGA-STAD (2.893 vs − 0.01; DRL TOP7) datasets but their regulation efficacy changes were not consistent. However, GIF was regulated by CEBPA in GSE54129 (− 3.412 vs 0.01; DRL TOP7). It was noticed that the DRLs of GIF were all ranked in TOP 30 in every dataset. According to the clinical information of TCGA-STAD, the GIF expression in different patient groups based on Neoplasm Histologic Grade were significantly different with Kruskal Wallis Test *p*-value 0.002 as shown in Additional file 1: Figure S4. We therefore suggest that GIF and its regulation are worthy of further investigation to elucidate its role in gastric carcinogenesis in diverse racial groups.

**Fig. 3:**
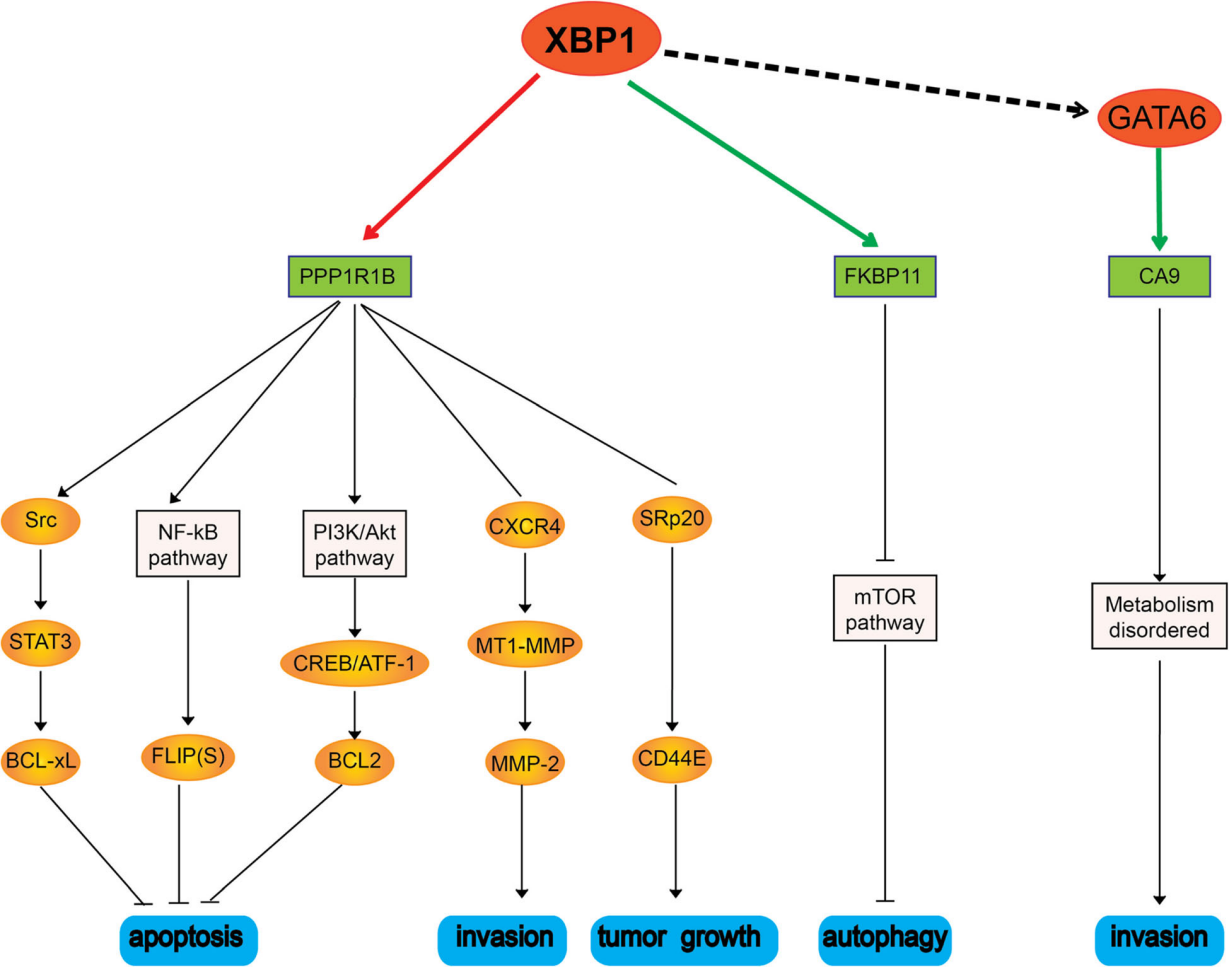
The hypotheses of dysfunctional regulation mechanisms of gastric carcinogenesis around XBP1. Red ellipses and green rectangles are TFs and targets obtained from our analysis respectively. The red line represents an increased positive regulation from normal to cancer, which also includes a decreased negative regulation; the green line represents a strengthened negative regulation from normal to cancer, which includes a decreased positive regulation as well. The dash line indicates the regulation relationship which exists in all three datasets while with differential regulation efficacy change. The orange ellipses, the white rectangles and the bottom blue rectangles are genes, pathway and cancer related biological processes obtained from the prior knowledge. As for the dark lines, ‘→’ indicates promoted effect; ‘—‘indicts protein-protein interaction; ‘-|’ indicates inhibited effect

XBP1 is the only overlapping transcription factor (TF) in the three top-100 gene lists across the three racial groups included in our analysis. Considering the crucial roles of TF in cancer progression, we put much attention to XBP1 although its DR rank and surrounding DRLs are all out of TOP-30 in every dataset and the mutation frequency of XBP1 is low (0.9%) in 588 gastric cancer samples in ICGC database (https://icgc.org/). By integrating our differential regulation analysis results to the prior knowledge, the dysfunctional regulation mechanisms underlying gastric carcinogenesis around XBP1 were proposed in Fig. 3. In all the three GC datasets, the positive regulation of PPP1R1B by XBP1 was increased from normal to cancer, and the negative regulation of FKBP11 by XBP1 was strengthened. The up-regulation of PPP1R1B was reported to inhibit apoptosis through NF-κB/FLIP(S) pathway [55] and promote cell invasion [56] and gastric tumorigenesis [57, 58]. The down-regulation of FKBP11 leads to the inhibition of autophagy through MTOR pathway and induces carcinogenesis [59]. In Chinese and Korean datasets (GSE54129 and GSE24375), the positive regulation of GATA6 by XBP1 was increased from normal to cancer, while it was decreased in American dataset (TCGA-STAD) during carcinogenesis. This might contribute to the differential gastric carcinogenesis between Asian and American. It is interesting that the positive regulation of CA9 by GATA6 was decreased in all three racial groups. This is consistent with the observation that Ca9 expression was frequently lost in gastric cancers in part by methylation [60], while contradictory to another report that the activity of Ca9 contributes to invasion and thus advanced tumor progression in a subset of gastric cancers [60–62].

## Discussion

In this work, we carried out a cross-racial integrative research on gastric cancer in terms of dysfunctional regulation mechanisms by implementing a novel differential networking meta-analysis framework. Differential regulation networking (DRN) analysis aims to identify the regulatory relationships relevant or even causative to phenotypic changes, which is challenging in the field of both computational and experimental biology. Since cancer has a nature of dysregulation mechanisms during carcinogenesis, the DRN analysis is helpful for deciphering differential regulation and differential networking underlying phenotypic changes in cancer. Even if lots of attention has been paid on traditional differential expression analysis in the past carcinogenesis studies, only in very recent years differential regulation networking analysis has become more and more widely applied [22, 23, 30]. With the transcriptomic data from cancer samples increasingly accumulated in the public domain, it is time to investigate the common dysfunctional regulation mechanisms of carcinogenesis at a broader systematic level. We therefore created a differential networking meta-analysis framework based on our previously developed metholologies, and applied it to three GC gene expression datasets corresponding to Chinese, Korean and American.

First of all, the high rank of cancer genes and drug targets in the three DRG lists (Table 2) as well as the basic statistics of the three conditional GRN pairs (Table 1) proved the rationality of the whole modelling strategy and the effectiveness of DR measure as expected. Since the DRN analysis module in the framework enables the discovery of novel regulators or regulatory relationships that have not yet been associated to the disease of interest [30, 31, 63–65], we hoped to find out novel dysregulation regulators and their related mechanisms underlying gastric carcinogenesis across different racial groups.

In our framework, DCGs were taken as the seed genes for differential network construction. As shown in Fig. 1, the DCGs between every two datasets are significantly overlapped, indicating a basically coherent profile of molecular interactions across Chinese, Korean, and American; meanwhile, we also noticed a closer overlap between Chinese and Korean, which was supported by previous reports [66, 67]. It is interesting that from the viewpoint of gene regulatory network (GRN), the three GRN pairs present significant differences from network topologies to DRG identities (Fig. 2). Furthermore, the consistency of highly ranked DRGs across three racial groups is quite limited as shown in Tables 3 and 4. The difference between racial groups could be associated with genetics, diet habits and other environmental factors. These observations strongly support the necessity of integrative studies in terms of differential regulation.

In order to decipher the common carcinogenesis across racial groups, we focus on those common DRGs across datasets. The DRGs commonly in at least two racial groups were presented in Table 4. Four genes (CEBPA, GATA6, GATA3 and GIF) were selected from TOP30 DRG lists, seven genes (GIF, XBP1, CEBPA, GATA6, DPP4, PTK7 and GATA3) were selected from TOP50 DRG lists, and 16 genes (GIF, XBP1, CEBPA, GATA6, DPP4, PTK7, GATA3, FOSB, EPAS1, CCND2, MALL, IRF1, SOX9, VILL, GALNT3 and LGALS4) were selected from TOP100 DRGs. Among the 16 cross-racial DRGs, all are cancer related according to ICGC and three genes (GATA3, SOX9 and CEBPA) have been regarded as cancer driver genes [68–71]. Functional enrichment analysis indicated that the 16 common cross-racial DRGs were enriched in the function of transcriptional regulation. We therefore propose that the highly ranked TFs among the 16 top common DRGs, including XBP1, FOSB, MRPL36, GATA3, GATA6, SOX9, POU2F1, IRF2, PTK7, EPAS1 and IRF1 (Table 4) are worthy of further investigation, especially on their roles of gene transcriptional regulation.

We then narrowed down our attention to GIF and XBP1 out of the 16 DRGs. Considering that GIF has been reported to be a GC prognosis biomarker [54, 72, 73], and GIF’s DRLs were all ranked in TOP 30 in every dataset, we suggest that GIF and its regulation are worthy of further investigation to elucidate its role in gastric carcinogenesis. The transcriptional regulation of GIF by GATA3 and CEBPA might be related to the racial specificity of GC. Although XBP1 has not been related to gastric cancer so far, it is the only overlapping transcription factor (TF) in the three top-100 DGR lists across Chinese, Korean and American. By integrating our DRN analysis results to prior knowledge, we proposed a hypothesis of dysfunctional regulation mechanisms for gastric carcinogenesis around XBP1 in Fig. 3. XBP1 and its DRLs seem to play crucial roles during gastric carcinogenesis across three racial groups.

The previous studies on the effects of racial factors on gastric cancer were mainly focused on statistics of clinical phenotypes, such as metastasis and survival [67, 74–76], and a few of literatures also reported genetic polymorphisms [66, 77]. By means of differential regulation analysis, our cross-racial meta-analysis of gastric cancer demonstrated the complexity and diversity in terms of differential networking, and made an insightful complement to these previous reports. The race specific and common cross-racial DRGs and DRLs are helpful to decipher the phenotypic differences among different racial GC groups from the perspective of transcriptional regulation, and helpful to discover universal or race specific drug targets or biomarkers.

The current work provides a differential networking meta-analysis framework which presents high cohesion and low coupling. This framework is extendable and adaptable to the studies on the regulation mechanisms of other cancer and even other phenotypic changes.

## Conclusions

Our study aimed at investigating the common dysregulation mechanisms of gastric carcinogenesis across racial groups from three Gastric Carcinoma datasets (GSE54129, GSE24375 and TCGA-STAD) for Chinese, Korean and American. We constructed a cross-racial integrative analysis framework from the viewpoint of differential regulation. The cross-racial meta-analysis of gastric cancer demonstrated the complexity and diversity of gastric carcinogenesis, and provided useful clues for understanding the common dysfunctional regulation mechanisms of gastric cancer progression. In addition, the differential networking meta-analysis framework presented here is extendable and adaptable to the studies on the regulation mechanisms of other cancer and even other phenotypic changes.

## Additional files

Additional file 1: Supplementary Figure and legends. **Figure S1.** Quality assessment of our data set (GSE54129). **Figure S2.** Expression level distribution of genes in three datasets. **Figure S3.** The differential networking meta-analysis framework. **Figure S4.** The GIF expression in different patient groups based on Neoplasm Histologic Grade. (DOCX 1497 kb)

Additional file 2: **Table S1.** DRN meta-analysis of gastric cancer, https://www.synapse.org/#!Synapse:syn10154163 (XLSX 98 kb)

## Abbreviations

DCEA: Differential CoExpression Analysis
DCG: Differential co-expression gene
DCL: Differential co-expression gene link
DRG: Differentially regulated gene
DRL: Differentially regulated gene link
DRN: Differential regulation networking
GC: Gastric Carcinoma
GRN: Gene regulatory network
STAD: Stomach adenocarcinoma
