# Supplementary material for: Differential networking meta-analysis of gastric cancer across Asian and American racial groups
Source: BMC Syst Biol. 2018 Apr 24;12(Suppl 4):51. doi: 10.1186/s12918-018-0564-z (PMC5998874; doi:10.1186/s12918-018-0564-z)
Supplement: Supplementary file 1 — Supplementary Figure and legends. Figure S1. Quality assessment of our data set (GSE54129). Figure S2. Expression level distribution of genes in three datasets. Figure S3. The differential networking meta-analysis framework. Figure S4. The GIF expression in different patient groups based on Neoplasm Histologic Grade. (DOCX 1497 kb) [file 12918_2018_564_MOESM1_ESM.docx]

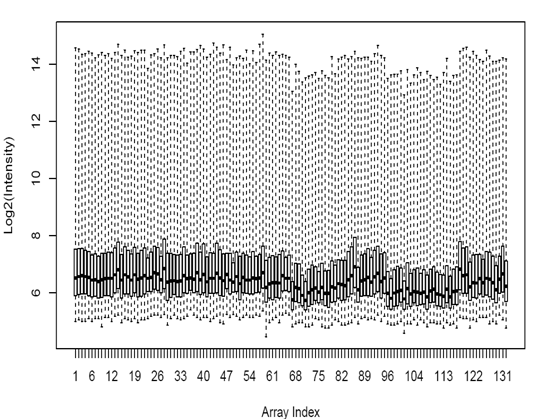

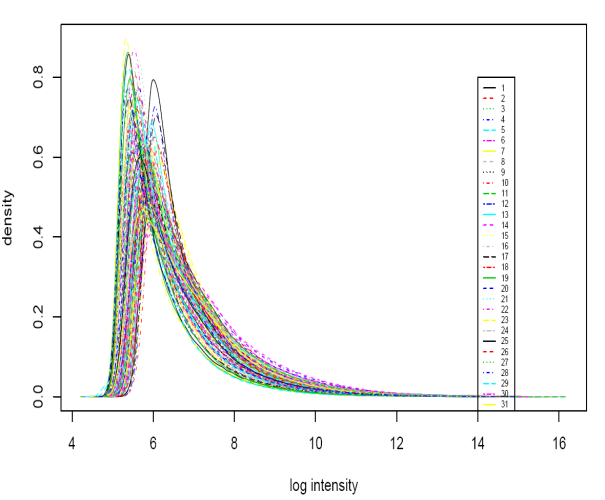

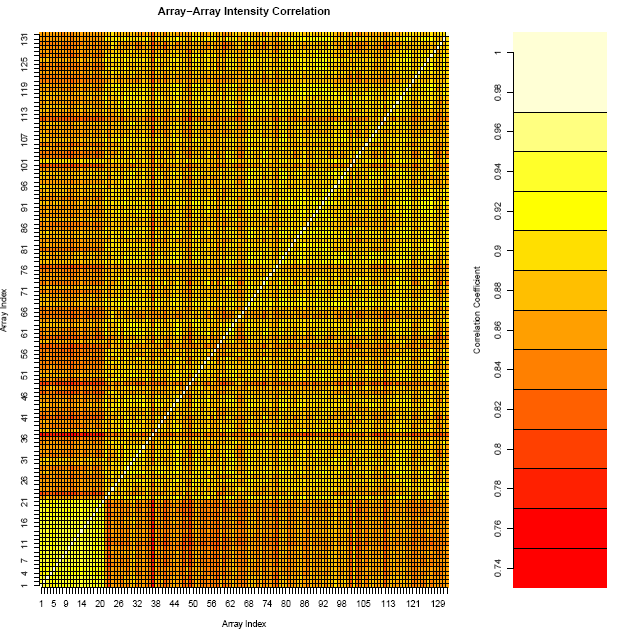


**A**

**B**

**C**

**Figure S1. Quality assessment of our data set (GSE54129)**. A) Distribution of log2 transformed expression level of genes in different samples. B) Density distribution of expression level of 132 samples. C) The correlations among 132 samples. The number ID 1 to 21 represent normal samples, others are cancer samples.


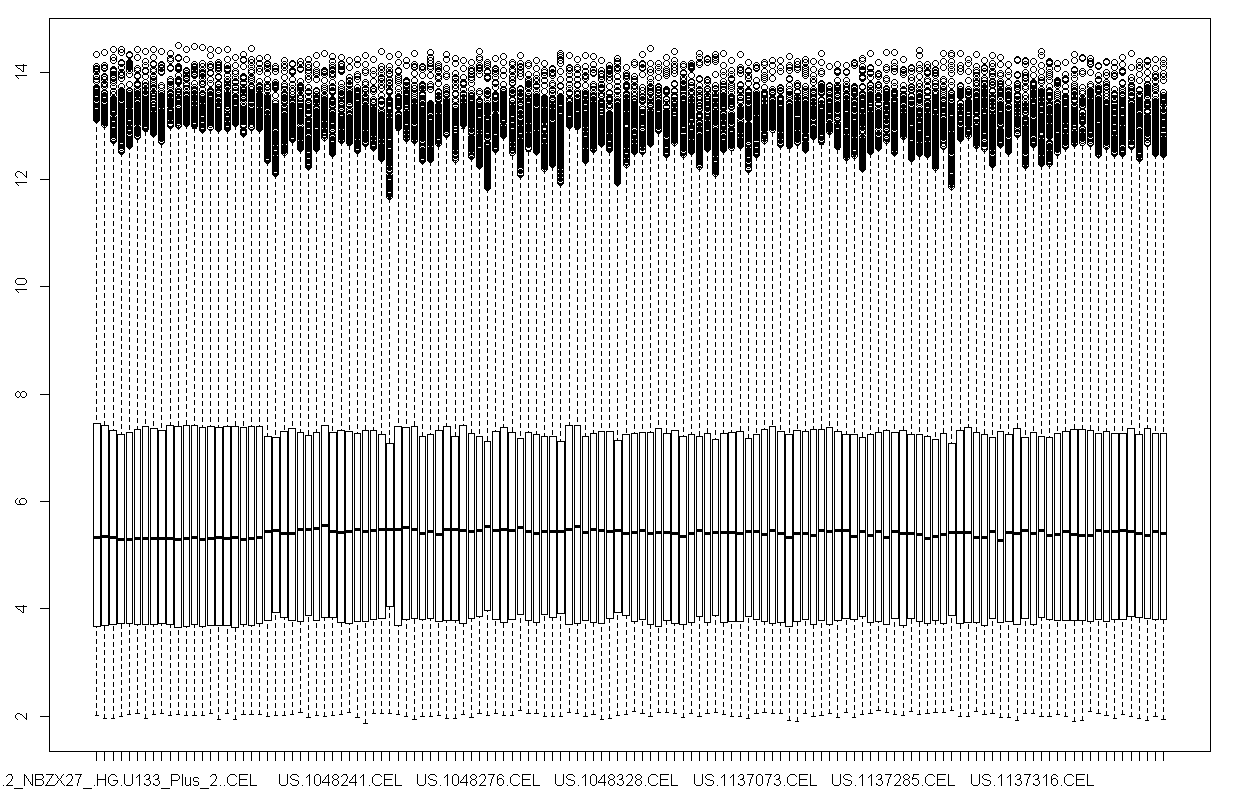

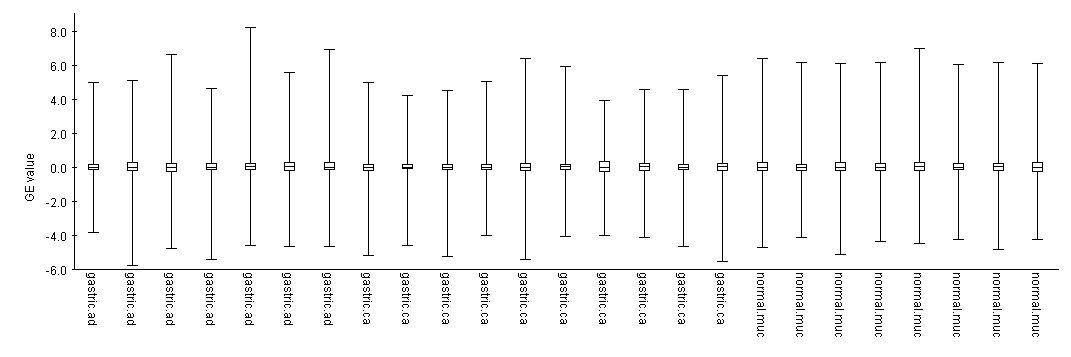

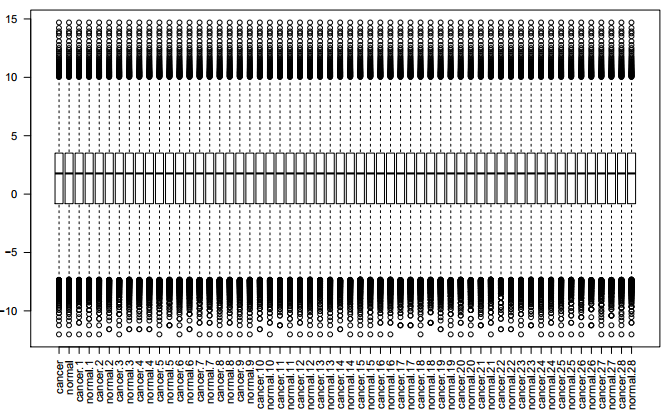


**A**

**B**

**C**

**Figure S2. Expression level distribution of genes in three datasets.** A) Our data set (GSE54129). B) GEO published dataset (GSE24375). C) TCGA-STAD dataset.


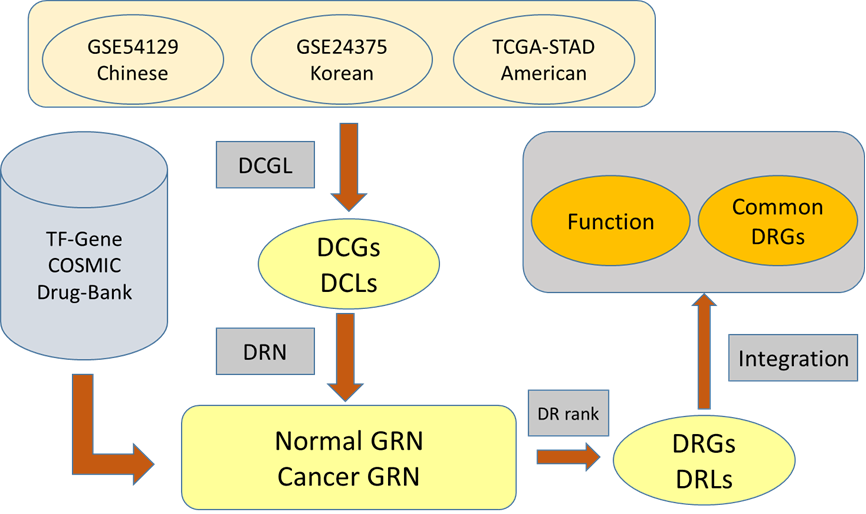


**Figure S3. The differential networking meta-analysis framework.**


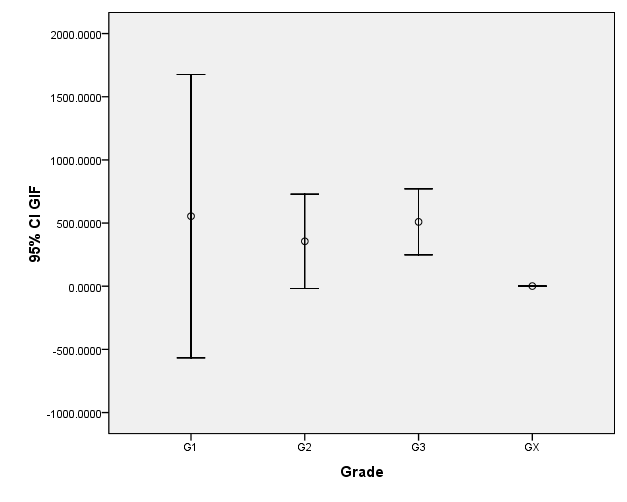


**Figure S4. The GIF expression in different patient groups based on Neoplasm Histologic Grade.** G1, G2, G3, and Gx groups have 9, 129, 225 and 6 patients, respectively.
